# Synergistic Activity of Nitroimidazole-Oxazolidinone Conjugates against Anaerobic Bacteria

**DOI:** 10.3390/molecules25102431

**Published:** 2020-05-22

**Authors:** Zhijun Zhuang, Dawei Wan, Jun Ding, Shijie He, Qian Zhang, Xiaomei Wang, Ying Yuan, Yu Lu, Charles Z. Ding, Anthony Simon Lynch, Anna M. Upton, Christopher B. Cooper, William A. Denny, Zhenkun Ma

**Affiliations:** 1 TenNor Therapeutics Limited, 218 Xinghu Street, Building B2, Suite 711, Suzhou Industrial Park, Suzhou 215123, China; zhijun.zhuang@tennorx.com (Z.Z.); dawei.wan@tennorx.com (D.W.); jun.ding@tennorx.com (J.D.); shijie.he@tennorx.com (S.H.); qian.zhang@tennorx.com (Q.Z.); xiaomei.wang@tennorx.com (X.W.); ying.yuan@tennorx.com (Y.Y.); 2 Department of Pharmacology, Beijing Tuberculosis and Thoracic Tumor Research Institute, Beijing Chest Hospital, Capital Medical University, 97 Ma Chang Street, Beijing 101149, China; luyu4876@hotmail.com; 3 WuXi AppTec. Co. Ltd., 288 Fute Zhong Road, Waigaoqiao Free Trade Zone, Shanghai 200131, China; charles_ding@wuxiapptec.com; 4 Janssen Research & Development LLC., 1400 McKean Road, Spring House, PA 18940, USA; alynch2@its.jnj.com; 5 Global Alliance for TB Drug Development, 40 Wall Street, New York, NY 10005, USA; anna.upton@tballiance.org (A.M.U.); christopher.cooper@tballiance.org (C.B.C.); 6 Auckland Cancer Society Research Centre, School of Medical Sciences, University of Auckland, Private Bag 92019, Auckland 1142, New Zealand; b.denny@auckland.ac.nz

**Keywords:** drug conjugates, anaerobic bacterium, synergy, nitroimidazole and oxazolidinone

## Abstract

The introductions of the bicyclic 4-nitroimidazole and the oxazolidinone classes of antimicrobial agents represented the most significant advancements in the infectious disease area during the past two decades. Pretomanid, a bicyclic 4-nitroimidazole, and linezolid, an oxazolidinone, are also part of a combination regimen approved recently by the US Food and Drug Administration for the treatment of pulmonary, extensively drug resistant (XDR), treatment-intolerant or nonresponsive multidrug-resistant (MDR) *Mycobacterium tuberculosis* (TB). To identify new antimicrobial agents with reduced propensity for the development of resistance, a series of dual-acting nitroimidazole-oxazolidinone conjugates were designed, synthesized and evaluated for their antimicrobial activity. Compounds in this conjugate series have shown synergistic activity against a panel of anaerobic bacteria, including those responsible for serious bacterial infections.

## 1. Introduction

**Figure 1:**
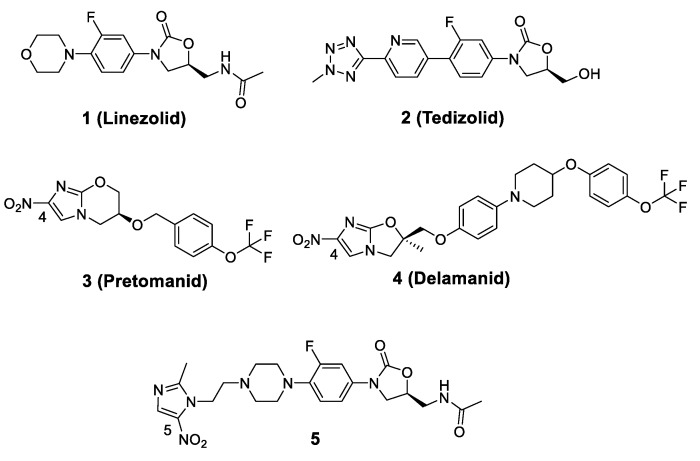
Structures of Linezolid, Tedizolid, Pretomanid, Delamanid and Conjugate Molecule **5**.

Oxazolidinones, as represented by linezolid (**1**) and tedizolid (**2**), and bicyclic 4-nitroimidazoles, as represented by pretomanid (**3**) and delamanid (**4**), are two relatively new classes of antimicrobial agents (Figure 1). Linezolid, the first oxazolidinone approved for clinical use, was introduced in 2000 for the treatment of Gram-positive bacterial infections, including those that are resistant to other classes of antibiotics [1]. This drug class inhibits bacterial protein synthesis by binding to rRNA on both the 30S and 50S ribosomal subunits and preventing the formation of a translation initiation complex. The first bicyclic 4-nitroimidazole, delamanid, was introduced in 2014 for the treatment of drug-resistant tuberculosis (TB). This drug class utilizes a deazaflavin-dependent nitroreductase (Ddn) to catalyze the bioreduction of the 4-nitroimidazole core, leading to the intracellular generation of reactive chemical species which are toxic to bacterial cells [2]. However, the primary mechanism of action of the bicyclic 4-nitroimidazole class against *M. tuberculosis* appeared to be different under aerobic and anaerobic conditions. Inhibition of mycolic acid synthesis appeared to be the main mechanism under aerobic conditions, while the generation of reactive nitrogen species and inhibition of energy metabolism appeared to be the main mechanism under anaerobic conditions [3].

Resistance to oxazolidinones and bicyclic 4-nitroimidazoles is relatively uncommon. Both drug classes have been used for the treatment of multidrug-resistant (MDR) and extensively drug resistant (XDR) TB. Recently, a three-drug combination containing both pretomanid and linezolid was approved by the US Food and Drug Administration for the treatment of pulmonary XDR, treatment-intolerant or nonresponsive MDR-TB [4].

Previously, a drug conjugation strategy was utilized to identify the dual-acting molecule TNP-2092 for the treatment of bacterial biofilm infections [5]. This strategy provides several advantages compared to drug combination therapy, including matched pharmacokinetics, tissue distribution and potential synergistic activity. Considering the importance of the oxazolidinone and the bicyclic 4-nitroimidazole classes, a series of oxazolidinone-nitroimidazole conjugate molecules was designed, synthesized and evaluated.

## 2. Results and Discussion

### 2.1. Chemistry

The safety and efficacy of the oxazolidinone and bicyclic 4-nitroimidazole classes have been validated clinically. The structure–activity relationships (SARs) of both drug classes have been extensively studied in the past, which serve as the foundation for the design of the conjugation molecules.

As a protein synthesis inhibitor, linezolid utilizes hydrogen bonding and hydrophobic interactions to bind to a binding domain located within the ribosomal peptidyltransferase center. A high resolution analysis of the of crystal structure of linezolid bound to the 50S ribosomal subunit indicated that the oxazolidinone ring and the acetamide group on the right side of the molecule are essential for the target interactions. The fluorophenyl moiety in the middle is also important. However, the morpholino ring on the left side does not appear to have significant interactions with the binding site, which is consistent with known SARs whose various structures can be used to substitute the morpholine group without significant loss of activity [6]. This position was therefore identified as the linking point for conjugation to a bicyclic 4-nitroimidazole core.

On the bicyclic 4-nitroimidazole side, the nitroimidazole group and its fused oxazine or oxazole ring are essential and directly responsible for the formation of intracellular reactive species via a bioreduction process. The stereochemistry of the substituents connected to the oxazine or oxazole ring also plays an important role for the antimicrobial activity. However, the structure of the substituents on the right-hand side are highly variable, and can tolerate many functional groups. This site was hence identified as the linking point for the bicyclic 4-nitroimidazole class.

**Scheme 1:**
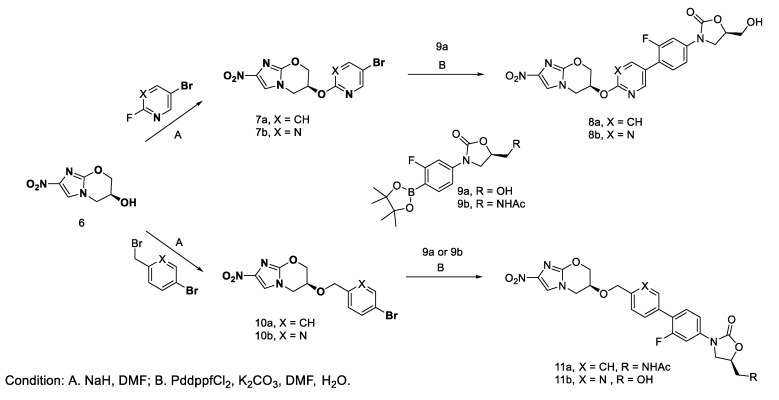
Synthesis of Conjugate Molecules **8a**–**b** and **11a**–**b**.

**Scheme 2:**
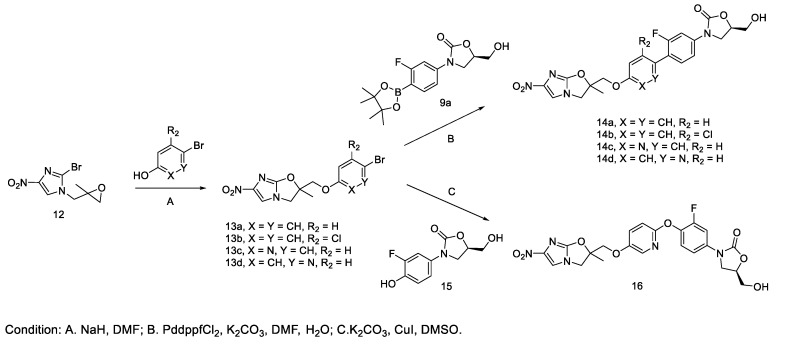
Synthesis of Conjugate Molecules **14a**–**d** and **16**.

**Scheme 3:**
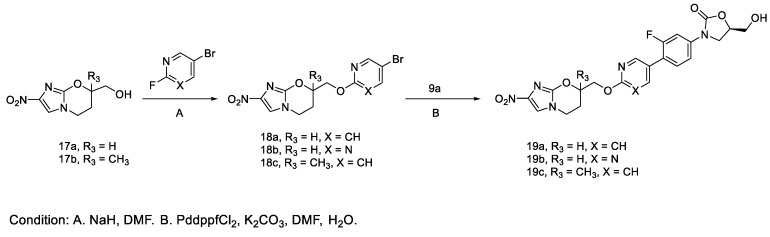
Synthesis of Conjugate Molecules **19a**–**c**.

A series of drug conjugate was therefore designed and synthesized by connecting the right-hand side of the bicyclic 4-nitroimidazole and the left-hand side of the oxazolidinone core through various linkers. The syntheses of these conjugate molecules are illustrated in Scheme 1, Scheme 2 and Scheme 3. Compound **5** (Figure 1) is a previously known oxazolidinone-metronidazole conjugate; it has not been evaluated for its activity against *M. tuberculosis* or anaerobic organisms [7]. Compound **5** was also prepared in the current study and its activity against *M. tuberculosis* isogenic mutant panel and anaerobic bacterial panel was evaluated.

The syntheses of the conjugate molecules formed by the oxazine fused nitroimidazole and the oxazolidinone core **8a**–**b** and **11a**–**b** are summarized in Scheme 1. The known (*S*)-2-nitro-6,7-dihydro-5*H*-imidazo[2,1-*b*][1,3]oxazin-6-ol core (**6**) was prepared according to literature procedures [8]. Intermediates **7a**–**b** and **10a**–**b** were prepared by aromatic or nucleophilic substitution reaction. Suzuki coupling with the oxazolidinone piece **9a** or **9b** provided conjugate molecules **8a**–**b** and **11a**–**b**.

The syntheses of the conjugate molecules formed by the oxazole-fused nitroimidazole and the oxazolidinone core **14a**–**d** and **16** are summarized in Scheme 2. The known racemate 2-bromo-1-((2-methyloxiran-2-yl)methyl)-4-nitro-1*H*-imidazole (**12**) was prepared according to a method described in the literature [9]. Intermediate **13a**–**d** were prepared by epoxide opening followed by intramolecular cyclization. Suzuki or copper-catalyzed coupling provided diastereomeric conjugate molecules **14a**–**d** and **16**. The ratio of the two diastereomers was not determined due to the fact that the two chiral centers in the molecules were distant from each other, and the two diastereomers could not be distinguished from each other spectroscopically. 

Scheme 3 summarizes the syntheses of a third series of conjugate molecules **19a**–**c**. This series contains the same oxazine fused nitroimidazole core as shown in Scheme 1, but connects to the oxazolidinones via a different linking point. The known racemate intermediates (2-nitro-6,7-dihydro-5*H*-imidazo[2,1-*b*][1,3]oxazin-7-yl)methanol (**17a**) and (7-methyl-2-nitro-6,7-dihydro-5*H*-imidazo[2,1-*b*][1,3]oxazin-7-yl)methanol (**17b**) were prepared according to a procedure described in the literature [10]. Aromatic substitution provided intermediates **18a**–**c**. Suzuki coupling with the oxazolidinone precursor **9a** provided diastereomeric conjugate molecules **19a**–**c**.

### 2.2. Mechanism of Action

The mechanisms of action of the precursor antibiotics of the conjugate molecules are well established. In order to understand whether the conjugate molecules synthesized in the current study maintained antibacterial activity and were enhanced by both precursor antibiotic classes, an isogenic panel of resistant mutant strains was prepared from wild-type strain *M. tuberculosis* H37Rv by stepwise resistance induction with linezolid or pretomanid (Table 1). Resistant mutant strains L1 and L3 were induced by linezolid with mutations on *rplC* gene encoding the 50S ribosomal protein L3. The minimum inhibitory concentrations (MICs) of linezolid against L1 and L3 were 4.80 and 8.18 µg/mL, respectively, as compared to 0.15 µg/mL against the wild-type H37Rv strain. The resistant mutant strains P1 and P3 were induced by pretomanid with mutations in the *ddn* gene encoding the deazoflavin-dependent nitroreductase. The MICs of pretomanid against P1 and P3 strains were >20 and >20 µg/mL respectively, as compared to 0.07 µg/mL for the H37Rv strain.

All four conjugate compounds listed in Table 1 (**8a**, **8b**, **14c** and **14d**) were highly active against the H37Rv strain with MICs similar to those of linezolid and pretomanid. More importantly, all compounds were significantly more potent than linezolid against the L1 and L3, strains and significantly more potent than pretomanid against the P1 and P3 mutant strains, indicating that the antimicrobial activity of the conjugate molecules benefits from contributions from both parental antibiotic pharmacophores.

Compounds **8a** and **8b** were formed by oxazine-fused 4-nitroimidazole and the oxazolidinone core. These compounds, particularly compound **8a**, appeared to be more potent against the linezolid resistant strains L1 and L3 than the pretomanid resistant strains P1 and P3, suggesting that the 4-nitroimidazole function contributes relatively more significantly to the antibacterial activity than the oxazolidonone function in these conjugate molecules. However, compounds **14c** and **14d**, formed by the oxazole fused 4-nitroimidazole and the oxazolidinone core, demonstrated balanced activity against the linezolid resistant strains L1 and L3 and the pretomanid resistant strains P1 and P3, suggesting that these conjugate molecules exhibit a balanced contribution from the two parental antibiotic pharmacophores.

### 2.3. Spectrum of Activity

Selected compounds from the current study were tested against a panel of representative pathogens (Table 2). All eight conjugate compounds tested showed a similar spectrum of activity as linezolid against the ESKAPE pathogens: *Enterococcus faecium* (Ef), *Staphylococcus aureus* (Sa), *Klebsiella pneumoniae* (Kp), *Acinetobacter baumannii* (Ab), *Pseudomonas aeruginosa* (Pa), and *Escherichia coli* (Ec). Specifically, these compounds were active against Ef and Sa, but inactive against Kp, Ac, Pa and Ec strains. Several compounds (**8a**, **8b**, **11a** and **14c**) were substantially more active than linezolid against Ef and Sa. All conjugate compounds were substantially more active than linezolid against the obligate anaerobic pathogen *Clostridium difficile* (Cd). The activities of these compounds were similar to those of metronidazole. Compound **11a,** with an acetamido group at the oxazolidinone ring, appeared to be more active than its hydroxyl counterpart **11b** against aerobic bacteria Ef and Sa. However, **11a** was similar or slightly less active than **11b** against anaerobic bacterium Cd. The different SARs between these two compounds against aerobic and anaerobic bacteria could be a result of the different mechanisms of action. In an aerobic bacterium, the activity is driven by the oxazolidinone pharmacophore, while in anaerobic bacteria, the activity is mainly driven by the nitroimidazole pharmacophore. The conjugate compounds were not cytotoxic, with IC_50s_ > 64 μg/mL for all compounds tested against the Vero cell-line.

### 2.4. Anaerobic Activity

The promising activity of the 4-nitromidazole-oxazolidinone conjugate series against the anaerobic pathogen *C. difficile* encouraged us to explore a broader panel of clinically important anaerobic bacteria (Table 3). The test panel included 13 strains of anaerobic bacteria and *Helicobacter pylori* (Hp), a Gram-negative microaerophilic bacterium. Seven of the 13 anaerobic bacterial strains were Gram-positive and the rest were Gram-negative. The full names of the anaerobic bacterial panel strains are listed in Table 4.

Metronidazole, a 5-nitroimidazole, is one of the most important drugs for the treatment of anaerobic bacterial infections. The activity of metronidazole against this panel was inconsistent, with virtually no activity against *Lactobacillus acidophilus* (La), *Peptostreptococcus micros* (Pm), *Propionibacterium acnes* (Pas) and *Veillonella parvula* (Vp). Pretomanid, a 4-nitroimidazole, exhibited a similar spectrum of activity as metronidazole against this panel, but with higher MICs. Linezolid was active against all the strains, with MICs ranging from 0.06 to 2 μg/mL against the 13 anaerobic bacteria. Interestingly, the 1:1 combination of linezolid and pretomanid was about one dilution less active than linezolid, virtually reflecting the activity of linezolid in the mixture. Pretomanid did not appear to contribute to the activity of the combination.

Compound **5**, a conjugate molecule between linezolid and metronidazole, a 5-nitroimidazole, appeared to be more potent than either of its precursor antibiotics against all strains tested, with MICs ranging from 0.06 to 1 μg/mL. The apparent synergistic effect of the 4-nitroimidazole-oxazolidione conjugate was more profound. The majority of the 12 compounds were significantly more potent than the combination of linezolid and pretomanid, with the exception of compound **16**. Several compounds (**8a**, **8b**, **11a**, **14c**, **19a**, **19b** and **19c**) were 10–100-fold more potent against the majority of the tested strains than the combination.

One of the limitations of the current study is that compounds **14a**–**d**, **16** and **19a**–**c** are diastereomers which may have an impact to the interpretation of the SARs, as one diastereomer may be more active than another.

The underlying mechanism for the apparent synergistic effect of this conjugate series is still unclear. A number of hypotheses are currently under consideration. The first is that conjugation of a nitroimidazole to the oxazolidinone simply makes the oxazolidinone more potent. The nitroimidazole group plays the role of a substituent that makes the oxazolidinone bind to the ribosomal RNA better. This hypothesis appears to be less plausible, as the synergistic effect was only observed with anaerobic bacteria. We did not observe the same level of improvement of potency against aerobic bacteria *Enterococcus faecium* and *Staphylococcus aureus* (Table 2). The second hypothesis is that the conjugate molecules bring additional nitroimidazole inside the cells by better penetration or avoidance of efflux. A third hypothesis is that the nitroimidazole moiety acts synergistically when conjugated to an oxazolidinone pharmacophore. The high binding affinity of the oxazolidinone moiety to ribosomes or ribosomal components could bring the reactive species generated from the nitroimidazole moiety into close affinity with the transcription/translation machinery and make them work more efficiently. This includes the possibility for irreversible covalent linking to the ribosome or other enzymes associated with intrinsic oxazolidinone resistance. The last two hypotheses are supported by the mechanism-of-action study which indicated that both the nitroimidazole and the oxazolidinone functions contribute the antibacterial activity inside a *M. tuberculosis* cell (Table 1).

## 3. Materials and Methods

### 3.1. Chemistry

General: Reference compounds linezolid, levofloxacin and metronidazole were purchased from ChemPacific (Baltimore, MD, USA) or Sigma-Aldrich (St. Louis, MO, USA). Pretomanid (PA-824) and compound **5** were prepared according to published procedures [7]. All other compounds were synthesized by TenNor Therapeutics (Suzhou, China).

All starting materials were either purchased from commercial sources or prepared according to published procedures. Operations involving moisture- and/or oxygen-sensitive materials were conducted under an atmosphere of nitrogen. Flash chromatography was performed using silica gel 60 as normal phase adsorbent or C18 silica gel as reverse phase adsorbent. Nuclear magnetic resonance (NMR) spectra were recorded on a Varian (Palo Alto, CA, USA) 400 MHz magnetic resonance spectrometer. ^1^H-NMR chemical shift is given in parts per million (δ) downfield from TMS. ^1^H-NMR information was tabulated in the following format: number of protons, multiplicity (s, singlet; d, doublet; t, triplet; q, quartet; m, multiplet; td, triplet of doublet; dt, doublet of triplet), coupling constant(s) (*J*) in hertz. The prefix app is occasionally applied in cases where the true signal multiplicity is unresolved, and prefix br indicates a broad signal. A high performance liquid chromatography (HPLC) analysis for the final compound was performed on an Agilent 1100 instrument using a Waters (Milford, MA, USA) Xterra RP18 column (5 μm, 4.6 mm × 250 mm) and gradient elution (solvent A, 20 mM NaH_2_PO4/acetonitrile, 60:40 *v*/*v*; solvent B, acetonitrile). HPLC purities for the final compound were ≥95%.

*(R)-3-(3-fluoro-4-(6-(((S)-2-nitro-6,7-dihydro-5H-imidazo[2,1-b][1,3]oxazin-6-yl)oxy)pyridin-3-yl)phenyl)-5-(hydroxymethyl)oxazolidin-2-one* (**8a**) [11]. To a solution of (*S*)-6-((5-bromopyridin-2-yl)oxy)-2-nitro-6,7-dihydro-5*H*-imidazo[2,1-*b*][1,3]oxazine (1.01 g, 2.96 mmol) and (*R*)-3-(3-fluoro-4-(4,4,5,5-tetramethyl-1,3,2-dioxaborolan-2-yl)phenyl)-5-(hydroxymethyl)-oxazolidin-2-onein DMF (30 mL) was added a solution of K_2_CO_3_ (0.90 g, 6.51 mmol) in water (1 mL) and the mixture was purged with nitrogen. Pd(PPh_3_)_4_ (0.17 g, 0.15 mmol) was then added and the mixture was warmed with stirring under nitrogen at 80 °C for 3 h. The solvents were completely removed under reduced pressure and the residue was partitioned between EtOAc and water. The extract was worked up and chromatographed on silica. Elution with EtOAc gave four fractions, then elution with (1:20) MeOH/DCM gave **8a** as a yellow powder (460 mg, 33%). This general procedure of Suzuki coupling reaction was also applied for the synthesis of **8b**, **11b**, **14a**–**d**, and **19a**–**c**. ^1^H-NMR (400 MHz, DMSO) δ 8.38 (s, 1H), 8.06(s, 1H), 7.94–7.91 (m, 1H), 7.63 (dt, *J* = 13.6, 2.4 Hz, 1H), 7.57 (d, *J* = 8.8 Hz, 1H), 7.46–7.43 (m, 1H), 6.97 (d, *J* = 9.2 Hz, 1H), 5.76 (s, 1H), 5.24 (t, *J* = 5.6 Hz, 1H), 4.74–4.67 (m, 2H), 4.48–4.38 (m, 2H), 4.13–4.09 (m, 1H), 3.86 (dd, *J* = 8.8, 6.0 Hz, 1H), 3.70–3.64 (m, 1H), 3.56–3.52 (m, 1H), 3.14 (d, *J* = 5.2 Hz, 1H). LC-MS (ESI): *m*/*z* = 472 (M + H)^+^.

*(R)-3-(3-fluoro-4-(2-(((S)-2-nitro-6,7-dihydro-5H-imidazo[2,1-b][1,3]oxazin-6-yl)oxy)pyrimidin-5-yl)phenyl)-5-(hydroxymethyl)oxazolidin-2-one* (**8b**) [11]. The title compound was prepared by following the same procedure as that described for the preparation of **8a**, except (*S*)-6-((5-bromopyrimidin-2-yl)oxy)-2-nitro-6,7-dihydro-5*H*-imidazo[2,1-*b*][1,3]oxazine was used instead of (*S*)-6-((5-bromopyridin-2-yl)oxy)-2-nitro-6,7-dihydro-5*H*-imidazo[2,1-*b*][1,3]oxazine. (580 mg, 41%). ^1^H-NMR (400 MHz, DMSO) δ 8.86 (s, 2H), 8.06 (s, 1H), 7.68 (d, *J* = 8.8 Hz, 1H), 7.66 (t, *J* = 2.8 Hz, 1H), 7.47 (dd, *J* = 8.8, 2.4 Hz, 1H), 5.74 (s, 1H), 5.23 (t, *J* = 5.6 Hz, 1H), 4.77–4.70 (m, 2H), 4.47 (s, 1H), 4.11 (t, *J* = 9.0 Hz, 1H), 3.86 (dd, *J* = 8.8, 6.0 Hz, 1H), 3.71–3.65 (m, 1H), 3.57–3.53 (m, 1H), 3.38–3.35 (m, 1H). LC-MS (ESI): *m*/*z* = 473 (M + H)^+^.

*N-(((S)-3-(2-fluoro-4′-((((S)-2-nitro-6,7-dihydro-5H-imidazo[2,1-b][1,3]oxazin-6-yl)oxy)methyl)-[1,1′-biphenyl]-4-yl)-2-oxooxazolidin-5-yl)methyl)acetamide* (**11a**) [11]. To a solution of (*S*)-6-((4-bromobenzyl)oxy)-2-nitro-6,7-dihydro-5*H*-imidazo[2,1-*b*][1,3]oxazine (70.4 mg, 0.21 mmol) and (*S*)-*N*-((3-(3-fluoro-4-(4,4,5,5-tetramethyl-1,3,2-dioxaborolan-2-yl)phenyl)-2-oxooxazolidin-5-yl) methyl)acetamide (86.0 mg, 0.227 mmol) in DMF (8 mL) was added a solution of Na_2_CO_3_ (57.0 mg, 0.538 mmol) in water (1 mL) and the mixture was purged with nitrogen. Pd(PPh_3_)_4_ (24.0 mg, 0.021 mmol) was then added and the mixture was warmed with stirring under nitrogen at 85 °C for 90 min. The solvents were completely removed under reduced pressure and the residue was partitioned between EtOAc and water. The extract was worked up and chromatographed on silica. Elution with EtOAc gave four fractions, then elution with (1:9) MeOH/EtOAc gave **11a** as an off-white powder. (59.4 mg, 44%). ^1^H-NMR (DMSO-D6, 400 MHz) δ 8.22 (t, *J* = 5.8 Hz, 1H), 8.07 (s, 1H), 7.60–7.50 (m, 4H), 7.39 (dd, *J* = 8.6, 2.2 Hz, 1H), 7.15 (d, *J* = 8.8 Hz, 2H), 5.29 (br s, 1H), 4.79–4.70 (m, 2H), 4.70–4.64 (m, 2H), 4.42 (dd, *J* = 13.8, 3.2 Hz, 1H), 4.35 (br d, *J* = 13.8 Hz, 1H), 4.16 (t, *J* = 9.0 Hz, 1H), 40.03 (M, 1H), 3.78 (dd, *J* = 9.2, 6.5 Hz, 1H), 3.43 (t, *J* = 5.5 Hz, 2H), 1.84 (s, 3H). LC-MS (ESI): *m*/*z* = 526 (M + H)^+^.

*(R)-3-(3-fluoro-4-(6-((((S)-2-nitro-6,7-dihydro-5H-imidazo[2,1-b][1,3]oxazin-6-yl)oxy)methyl)pyridin-3-yl)phenyl)-5-(hydroxymethyl)oxazolidin-2-one* (**11b**) [11]. The title compound was prepared by following the same procedure as that described for the preparation of **8a** except (*S*)-6-((5-bromopyridin-2-yl)methoxy)-2-nitro-6,7-dihydro-5*H*-imidazo[2,1-*b*][1,3]oxazine was used instead of (*S*)-6-((5-bromopyridin-2-yl)oxy)-2-nitro-6,7-dihydro-5*H*-imidazo[2,1-*b*][1,3]oxazine. (360 mg, 38%). ^1^H-NMR (400 MHz, DMSO) δ 8.68 (s, 1H), 8.04 (s, 1H), 7.96 (dt, *J* = 8.8, 1.6 Hz, 1H), 7.65 (dd, *J* = 13.6, 2.4 Hz, 1H), 7.60 (d, *J* = 8.8 Hz, IH), 7.46 (dd, *J* = 8.4, 2.8 Hz, 2H), 5.74 (s, 1H), 5.24 (t, *J* = 5.6 Hz, 1H), 4.77–4.69 (m, 3H), 4.48 (d, *J* = 12.0 Hz, 1H), 4.35–4.22 (m, 3H), 4.12 (t, *J* = 9.2 Hz, 1H), 3.86 (dd, *J* = 8.8, 5.6 Hz, 1H), 3.70–3.65 (m, 1H), 3.58–3.52 (m, 1H), 3.16–3.12 (m, 1H). LC-MS (ESI): *m*/*z* = 486 (M + H)^+^.

*(5R)-3-(2-fluoro-4′-((2-methyl-6-nitro-2,3-dihydroimidazo[2,1-b]oxazol-2-yl)methoxy)-[1,1′-biphenyl]-4-yl)-5-(hydroxymethyl)oxazolidin-2-one* (**14a**). The title compound was prepared by following the same procedure as that described for the preparation of **8a**, except (*S*)-6-((5-bromopyridin-2-yl)methoxy)-2-nitro-6,7-dihydro-5*H*-imidazo[2,1-*b*][1,3]oxazine was used instead of (*S*)-6-((5-bromopyridin-2-yl)oxy)-2-nitro-6,7-dihydro-5*H*-imidazo[2,1-*b*][1,3]oxazine (2.87 g, 56.3%). ^1^H-NMR (400 MHz, DMSO) δ 8.14 (s, 1H), 7.67–7.26 (m, 5H), 6.96 (d, *J* = 8.8 Hz, 2H), 5.20 (t, *J* = 5.6 Hz, 1H), 4.69 (td, *J* = 9.3, 3.6 Hz, 1H), 4.39–4.28 (m, 3H), 4.17 (d, *J* = 11.0 Hz, 1H), 4.07 (t, *J* = 9.0 Hz, 1H), 3.82 (dd, *J* = 8.8, 6.3 Hz, 1H), 3.65 (ddd, *J* = 12.2, 5.4, 3.2 Hz, 1H), 3.58–3.48 (m, 1H), 1.66 (s, 3H). LC-MS (ESI): *m*/*z* = 485 (M + H)^+^.

*(5R)-3-(2′-chloro-2-fluoro-4′-((2-methyl-6-nitro-2,3-dihydroimidazo[2,1-b]oxazol-2-yl)methoxy)-[1,1′-biphenyl]-4-yl)-5-(hydroxymethyl)oxazolidin-2-one* (**14b**). The title compound was prepared by following the same procedure as that described for the preparation of **8a**, except 2-((4-bromo-3-chlorophenoxy)methyl)-2-methyl-6-nitro-2,3-dihydroimidazo[2,1-*b*]oxazole was used instead of (*S*)-6-((5-bromopyridin-2-yl)oxy)-2-nitro-6,7-dihydro-5*H*-imidazo[2,1-*b*][1,3]oxazine (346 mg, 66.7%). ^1^H-NMR (400 MHz, DMSO) δ 8.13 (s, 1H), 7.55 (dd, *J* = 12.5, 2.0 Hz, 1H), 7.37 (dd, *J* = 8.6, 2.0 Hz, 1H), 7.28 (dd, *J* = 15.5, 8.5 Hz, 2H), 7.13 (d, *J* = 2.5 Hz, 1H), 6.93 (dd, *J* = 8.6, 2.5 Hz, 1H), 5.20 (t, *J* = 5.6 Hz, 1H), 4.69 (dd, *J* = 9.1, 5.6 Hz, 1H), 4.41–4.29 (m, 3H), 4.16 (d, *J* = 11.0 Hz, 1H), 4.08 (t, *J* = 9.0 Hz, 1H), 3.82 (dd, *J* = 8.9, 6.1 Hz, 1H), 3.69–3.60 (m, 1H), 3.58–3.49 (m, 1H), 1.65 (s, 3H). LC-MS (ESI): *m*/*z* = 567 (M + H)^+^.

*(5R)-3-(3-fluoro-4-(6-((2-methyl-6-nitro-2,3-dihydroimidazo[2,1-b]oxazol-2-yl)methoxy)pyridin-3-yl)phenyl)-5-(hydroxymethyl)oxazolidin-2-one* (**14c**). The title compound was prepared by following the same procedure as that described for the preparation of **8a**, except 2-(((5-bromopyridin-2-yl)oxy)methyl)-2-methyl-6-nitro-2,3-dihydroimidazo[2,1-*b*]oxazole was used instead of (*S*)-6-((5-bromopyridin-2-yl)oxy)-2-nitro-6,7-dihydro-5*H*-imidazo[2,1-*b*][1,3]oxazine (3.5 g, 51.5%). ^1^H-NMR (400 MHz, DMSO ) δ 8.34 (s, 1H), 8.15 (s, 1H), 7.94–7.87 (m, 1H), 7.68–7.55 (m, 2H), 7.45 (dd, *J* = 8.6, 2.1 Hz, 1H), 6.86 (d, *J* = 8.6 Hz, 1H), 5.22 (t, *J* = 5.6 Hz, 1H), 4.74 (td, *J* = 5.8, 2.7 Hz, 1H), 4.63 (s, 2H), 4.41 (d, *J* = 11.0 Hz, 1H), 4.25–4.09 (m, 2H), 3.88 (dd, *J* = 8.9, 6.2 Hz, 1H), 3.70 (ddd, *J* = 12.3, 5.5, 3.4 Hz, 1H), 3.58 (ddd, *J* = 12.3, 5.7, 4.1 Hz, 1H), 1.71 (s, 3H). LC-MS (ESI): *m*/*z* = 486 (M + H)^+^.

*(5R)-3-(3-fluoro-4-(5-((2-methyl-6-nitro-2,3-dihydroimidazo[2,1-b]oxazol-2-yl)methoxy)pyridin-2-yl)phenyl)-5-(hydroxymethyl)oxazolidin-2-one* (**14d**). The title compound was prepared by following the same procedure as that described for the preparation of **8a**, except 2-(((6-bromopyridin-3-yl)oxy)methyl)-2-methyl-6-nitro-2,3-dihydroimidazo[2,1-*b*]oxazole was used instead of (*S*)-6-((5-bromopyridin-2-yl)oxy)-2-nitro-6,7-dihydro-5*H*-imidazo[2,1-*b*][1,3]oxazine (280 mg, 57.7%). ^1^H-NMR (400 MHz, DMSO) δ 8.32 (d, *J* = 2.9 Hz, 1H), 8.16 (d, *J* = 17.1 Hz, 1H), 7.90 (t, *J* = 9.0 Hz, 1H), 7.69 (d, *J* = 7.6 Hz, 1H), 7.58 (dd, *J* = 14.2, 1.9 Hz, 1H), 7.48–7.38 (m, 2H), 5.21 (t, *J* = 5.6 Hz, 1H), 4.70 (dd, *J* = 9.0, 5.7 Hz, 1H), 4.40 (dt, *J* = 11.0, 9.4 Hz, 3H), 4.17 (d, *J* = 11.0 Hz, 1H), 4.08 (t, *J* = 9.0 Hz, 1H), 3.83 (dd, *J* = 8.7, 6.3 Hz, 1H), 3.65 (ddd, *J* = 12.2, 5.2, 3.4 Hz, 1H), 3.59–3.49 (m, 1H), 1.67 (s, 3H). LC-MS (ESI): *m*/*z* = 486 (M + H)^+^.

*(5R)-3-(3-fluoro-4-((5-((2-methyl-6-nitro-2,3-dihydroimidazo[2,1-b]oxazol-2-yl)methoxy)pyridin-2-yl)oxy)phenyl)-5-(hydroxymethyl)oxazolidin-2-one* (**16**).

*2-(((5-bromopyridin-2-yl)oxy)methyl)-2-methyl-6-nitro-2,3-dihydroimidazo[2,1-b]oxazole* (532 mg, 1.5 mmol), (*R*)-3-(3-fluoro-4-hydroxyphenyl)-5-(hydroxymethyl)oxazolidin-2-one (376 mg, 1.65 mmol), Potassium carbonate (622 mg, 4.5 mmol), Cuprous iodide (286 mg, 1.5 mmol) and N,N,N′,N′-Tetramethylethylenediamine (TMEDA) (174 mg, 1.5 mmol) were added in N,N-dimethylformamide (5 mL). The mixture was stirred at 90 °C for 12 h. The mixture was cooled to room temperature, then poured into water (50 mL), the solvent was filtered and the filtered solid was pump dried. The crude product was further purified by silica chromatography column (DCM:MeOH = 100:2) to give (5*R*)-3-(3-fluoro-4-((5-((2-methyl-6-nitro-2,3-dihydroimidazo-[2,1-*b*]oxazol-2-yl)methoxy)pyridin-2-yl)oxy)phenyl)-5-(hydroxymethyl)oxazolidin-2-one(200 mg, 26.6%). ^1^H NMR (500 MHz, DMSO) δ 8.29 (s, 1H), 8.05 (s, 1H), 7.88 (d, *J* = 8.4 Hz, 1H), 7.64–7.50 (m, 2H), 7.41 (d, *J* = 8.5 Hz, 1H), 6.92 (d, *J* = 8.5 Hz, 1H), 4.70 (s, 1H), 4.48 (s, 2H), 4.23–4.01 (m, 3H), 3.83 (t, *J* = 7.3 Hz, 1H), 3.65 (d, *J* = 12.4 Hz, 1H), 3.53 (d, *J* = 12.2 Hz, 1H), 2.40–2.29 (m, 1H), 2.22–2.09 (m, 1H), 1.46 (s, 3H). LC-MS (ESI): *m*/*z* = 502 (M + H)^+^.

*(5R)-3-(3-fluoro-4-(6-((2-nitro-6,7-dihydro-5H-imidazo[2,1-b][1,3]oxazin-7-yl)methoxy)pyridin-3-yl)phenyl)-5-(hydroxymethyl)oxazolidin-2-one* (**19a**). The title compound was prepared by following the same procedure as that described for the preparation of **8a**, except 7-(((5-bromopyridin-2-yl)oxy)methyl)-2-nitro-6,7-dihydro-5*H*-imidazo[2,1-*b*][1,3]oxazine was used instead of (*S*)-6-((5-bromopyridin-2-yl)oxy)-2-nitro-6,7-dihydro-5*H*-imidazo[2,1-*b*][1,3]oxazine (162 mg, 56.3%). ^1^H-NMR (400 MHz, DMSO) δ 8.32 (s, 1H), 8.06 (s, 1H), 7.91 (d, *J* = 8.2 Hz, 1H), 7.58 (dd, *J* = 22.0, 11.8 Hz, 2H), 7.42 (d, *J* = 8.0 Hz, 1H), 6.97 (d, *J* = 8.5 Hz, 1H), 5.21 (t, *J* = 5.4 Hz, 1H), 4.91 (s, 1H), 4.71 (d, *J* = 2.9 Hz, 1H), 4.57 (dt, *J* = 12.1, 7.7 Hz, 2H), 4.21–4.01 (m, 3H), 3.89–3.80 (m, 1H), 3.66 (d, *J* = 11.5 Hz, 1H), 3.60–3.49 (m, 1H), 2.29 (d, *J* = 13.4 Hz, 1H), 2.18 (dd, *J* = 15.1, 9.6 Hz, 1H). LC-MS (ESI): *m*/*z* = 486 (M + H)^+^.

*(5R)-3-(3-fluoro-4-(2-((2-nitro-6,7-dihydro-5H-imidazo[2,1-b][1,3]oxazin-7-yl)methoxy)pyrimidin-5-yl)phenyl)-5-(hydroxymethyl)oxazolidin-2-one* (**19b**). The title compound was prepared by following the same procedure as that described for the preparation of **8a**, except 7-(((5-bromopyrimidin-2-yl)oxy)methyl)-2-nitro-6,7-dihydro-5*H*-imidazo[2,1-*b*][1,3]oxazine was used instead of (*S*)-6-((5-bromopyridin-2-yl)oxy)-2-nitro-6,7-dihydro-5*H*-imidazo[2,1-*b*][1,3]oxazine (674 mg, 50.3%). ^1^H-NMR (400 MHz, DMSO) δ 8.81 (d, *J* = 0.9 Hz, 2H), 8.07 (s, 1H), 7.70–7.62 (m, 2H), 7.48–7.41 (m, 1H), 5.22 (t, *J* = 5.6 Hz, 1H), 5.01–4.90 (m, 1H), 4.76–4.61 (m, 3H), 4.20–4.06 (m, 3H), 3.85 (dd, *J* = 8.8, 6.3 Hz, 1H), 3.66 (ddd, *J* = 12.3, 5.2, 3.4 Hz, 1H), 3.54 (ddd, *J* = 12.4, 5.6, 4.1 Hz, 1H), 2.30 (dd, *J* = 11.9, 2.4 Hz, 1H), 2.24–2.10 (m, 1H). LC-MS (ESI): *m*/*z* = 487 (M + H)^+^.

*(5R)-3-(3-fluoro-4-(6-((7-methyl-2-nitro-6,7-dihydro-5H-imidazo[2,1-b][1,3]oxazin-7-yl)methoxy)pyridin-3-yl)phenyl)-5-(hydroxymethyl)oxazolidin-2-one* (**19c**). The title compound was prepared by following the same procedure as that described for the preparation of **8a** except 7-(((5-bromopyridin-2-yl)oxy)methyl)-7-methyl-2-nitro-6,7-dihydro-5*H*-imidazo[2,1-*b*][1,3]oxazine was used instead of (*S*)-6-((5-bromopyridin-2-yl)oxy)-2-nitro-6,7-dihydro-5*H*-imidazo[2,1-*b*][1,3]oxazine (260 mg, 68.6%). ^1^H-NMR (500 MHz, DMSO) δ 8.29 (s, 1H), 8.05 (s, 1H), 7.88 (d, *J* = 8.4 Hz, 1H), 7.64–7.50 (m, 2H), 7.41 (d, *J* = 8.5 Hz, 1H), 6.92 (d, *J* = 8.5 Hz, 1H), 4.70 (s, 1H), 4.48 (s, 2H), 4.11 (dt, *J* = 17.0, 7.2 Hz, 3H), 3.83 (t, *J* = 7.3 Hz, 1H), 3.65 (d, *J* = 12.4 Hz, 1H), 3.53 (d, *J* = 12.2 Hz, 1H), 2.40–2.29 (m, 1H), 2.22–2.09 (m, 1H), 1.46 (s, 3H). LC-MS (ESI): *m*/*z* = 500 (M + H)^+^.

### 3.2. Biology

#### 3.2.1. Bacterial Strains 

The bacterial strains used in the current study are listed in Table 4. Strains in the *Mycobacterium tuberculosis* isogenic resistant mutant panel used for the mechanism of action study were generated and tested at Beijing Tuberculosis and Thoracic Tumor Research Institute (BTTTRI, Bejing, China). Strains of the spectrum panel were all ATCC stains, and testing was conducted at HD Biosciences (Shanghai, China). Strains of the anaerobic bacterial panel were ATCC strains, and the test was conducted at Micomyx Inc. (Kalamazoe, MI, USA).

#### 3.2.2. Generation of Isogenic Mutant Panel

The methods to induce and characterize the Mtb isogenic mutant strains resistant to pretomanid or linezolid have been described previously [12,13]. Briefly, 200 µL of 10^4^–10^5^ CFU/mL of *M. tuberculosis* H37Rv (ATCC27294) in 7H9 liquid culture medium at logarithmic growth phase were evenly spread on a 7H11 solid medium containing 1× MIC (0.07 µg/mL) of pretomanid and blank control medium. After sealing, the plates were incubated at 37 °C under 5% CO_2_ for 3 to 4 weeks. Single colonies with good growth were collected from the drug-containing solid medium, milled and diluted to a concentration of 10^4^–10^5^ CFU/mL, and then inoculated onto 7H11 solid medium with pretomanid at 2× MIC (0.15 μg/mL) and blank control medium. The plates were then incubated under the same conditions for another 3 to 4 weeks to observe the growth of single colonies. A single colony with robust growth was collected from the drug-containing medium and further subcultured on the drug-containing solid medium with a 2-fold increase of concentration of pretomanid until the selection of a single colonies that grew well on solid medium with pretomanid concentration of 16× MIC. The phenotypic confirmation of the pretomanid resistant mutant strains was performed by the Alamar Blue double dilution method [14]. The genotypic change of the resistant mutant strains were confirmed to be the T265C point mutation (TAC→CAC, Y89H) in the *ddn* gene. A similar sequential drug selection method was applied to obtain linezolid resistant mutant trains. The genotypic change of the linezolid resistant mutant strains was found to be the point mutation of the *rplC* (T460C).

#### 3.2.3. Media 

Media were prepared according to guidelines from CLSI. The test medium used for the anaerobic bacterial was supplemented Brucella broth (BD, Lot No. 6278735) containing 5 μg/mL hemin (Sigma (St. Louis, MO. USA), Lot No. SLBC4685V), 1 mg/mL Vitamin K1 (Sigma, Lot No. MKBN5958V), and 5% (*v*/*v*) laked horse blood (LHB, Cleveland Scientific (Bath, OH, USA), Lot No. 385663). Brucella broth (BD) supplemented with 10% fetal bovine serum (FBS, Gibco, Lot No. 1709261) was used for testing of *H. pylori*. Cation-adjusted Mueller Hinton Broth (CAMHB) media was used for the spectrum determination against the spectrum panel. Difco Middlebrook (Waltham, MA, USA) 7H9 Broth (Catalog No. 271310) supplemented with 0.2% (*v*/*v*) glycerol, 0.05% Tween 80, and 10% (*v*/*v*) albumin-dextrosecatalase (BBL Middlebrook (Waltham, MA, USA) ADC Enrichment, Catalog No. 212352) (7H9-ADC-T) was used for the MIC assay against the *M. tuberculosis* isogenic resistant mutant panel.

#### 3.2.4. Minimum Inhibitory Concentration Testing

The MIC test against the spectrum panel was conducted at HB Biosciences, following the broth microdilution method per CLSI guidance. The six ESKAPE ATCC strains were recovered in Trypticase Soy Agar (TSA) plates and tested with drug in CAMHB. Anaerobic bacterial *C. difficile* strains were recovered on TSA agar, grown and tested with compounds in Blucella broth with 5 µg/mL chlorhematin and 10 µg/mL vitamin K_1_ in an anaerobic chamber.

The MIC assay against the Mtb isogenic resistant mutant strains was performed at BTTTRI by the microplate Alamar blue assay [14]. Pretomanid, linezolid and metronidazole were used as comparators. The H37Rv strain and its derived isogenic resistant mutant strains were grown for 1–2 weeks at 37 °C and adjusted to a turbidity of McFarland 1 at 10^7^ CFU/mL and diluted 1:20. Twofold dilutions of testing compounds and comparators were prepared in 7H9-ADC-TG in volumes of 100 µL in 96-well, black, clear-bottom microplates (BD Biosciences, Franklin Lakes, NJ, USA). Bacterial cells (100 µL containing 2 × 10^5^ CFU) was added, yielding a final testing volume of 200 µL. The plates were incubated at 37 °C, and on day 7, 12.5 µL of 20% Tween 80 and 20 µL of Alamar blue were added to all wells. After incubation at 37 °C for 16 to 24 h, the fluorescence was read at an excitation of 530 nm and an emission of 590 nm. The MIC was defined as the lowest concentration effecting a reduction in fluorescence of ≥90% relative to the mean of replicate bacterium-only controls.

The MIC assay against anaerobic bacterial strains was performed at Micromyx by following the procedure described by CLSI [15,16,17]. A standardized inoculum of each organism was prepared as per CLSI methods. Colonies were picked from the primary plate and a suspension was prepared to equal to a 0.5 McFarland turbidity standard. Anaerobic suspensions were diluted 1:10 in Brucella broth with 5% laked horse blood, and each well was inoculated with 10 μL using a multichannel pipette in the Bactron anaerobe chamber, resulting in a final cell density of approximately 5 × 10^5^ CFU/mL (5 × 10^4^ CFU/mL for *Clostridium* spp.). For the *H. pylori* strain, colonies were picked from the primary plate and a suspension was prepared to equal a 2.0 McFarland turbidity standard. Suspensions were diluted 1:15 in Brucella broth with 10% FBS, and then transferred to compartments of sterile reservoirs divided by length (Beckman Coulter). The Biomek 2000 (Brea, CA, United States) was used to inoculate the plates. Daughter plates were placed on the Biomek 2000 work surface in reverse orientation so that plates were inoculated from low to high drug concentration. The Biomek 2000 delivered 10 μL of standardized inoculum into each well of the appropriate daughter plate for an additional 1:20 dilution. Anaerobe plates were placed in an anaerobic box with GasPak sachets (BD), and were incubated anaerobically for 46–48 h at 35–37 °C. *H. pylori* was incubated for 72 h in a microaerophilic atmosphere using boxes with GasPak EZ Campy sachets (BD) prior to reading. After incubation, plates were viewed from the bottom using a plate viewer. An uninoculated solubility control plate was observed for evidence of drug precipitation. MIC values were read and recorded as the lowest concentration of drug that inhibited the visible growth of the organism.

#### 3.2.5. Cytotoxicity Assay 

The cytotoxicity assay followed the previously published protocol [14]. Briefly, Vero cells in RPMI 1640 medium supplemented with 10% fetal bovine serum (FBS) were incubated in a humidified atmosphere of 5% CO_2_ at 37 °C to reach confluent, and then diluted to 4 × 10^5^ cells/mL. Threefold serial dilutions of the stock solutions resulted in final concentrations of 64 to 0.26 µg/mL in a final volume of 100 µL. Cytotoxicity testing was performed in a transparent 96-well microplate. After incubation at 37 °C for 48 h, the medium was removed, and the monolayers were washed twice with 100 µL of warm Hanks balanced salt solution (HBSS). Warm medium and freshly made methyl-thiazolyldiphenyl-tetrazolium bromide (MTT) were added to each well, and then the plates were incubated for 4 h, after which the absorbance was determined at 492 nm.

## 4. Conclusions

A series of 4-nitroimidazole and oxazolidione conjugate molecules was designed, synthesized and evaluated based on previous structure-activity relationship information. The dual mechanism of action of this series was demonstrated against *Mycobacterium tuberculosis* by utilizing an isogenic mutant panel resistant to either pretomanid or linezolid. Compounds in the series are highly active against a panel of clinically important anaerobic bacteria. A strong synergistic effect was observed compared to the combination of linezolid and pretomanid. It can be concluded that the nitroimidazole-oxazolidinone conjugate molecules hold potential for the treatment of anaerobic bacterial infections.

## Figures and Tables

**Table 1:** Minimum inhibitory concentrations of selected conjugate molecules against isogenic resistant mutant strains of *M. tuberculosis*.

Compounds	*M. tuberculosis* Isogenic Mutant Panel MIC (μg/mL)				
	H37Rv	L1	L3	P1	P3
**Linezolid**	0.15	4.80	8.18	0.29	0.31
**Pretomanid**	0.07	0.15	0.24	>20	>20
**8a**	0.06	1.95	1.78	6.48	5.96
**8b**	0.20	0.91	1.87	1.04	3.30
**14c**	0.08	1.84	0.81	1.77	1.80
**14d**	0.33	1.88	1.46	1.93	2.94

**Table 2:** Minimum inhibitory concentrations (MIC) of selected conjugate molecules against representative strains from seven major pathogen classes.

Compounds	Representative of Major Pathogens MIC (μg/mL)							Vero IC_50_ μg/mL
	Ef ATCC 708221	Sa ATCC 29213	Kp ATCC 43816	Ab ATCC 19606	Pa ATCC 27853	Ec ATCC 25922	Cd ATCC 43255	
**Linezolid**	4	8	>64	>64	>64	>64	4	>64
**Levofloxacin**	>16	0.25	0.063	0.5	1	<0.016	4	-
**Metronidazole**	>64	64	>64	>64	>64	>64	0.25	-
**8a**	0.5	1	>64	>64	>64	>64	0.125	>64
**8b**	2	2	>64	>64	>64	>64	0.25	>64
**11a**	1	2	>64	>64	>64	>64	0.25	-
**11b**	4	8	>64	>64	>64	>64	0.125	>64
**14a**	4	4	>64	>64	>64	>64	0.25	>64
**14b**	8	16	>64	>64	>64	>64	0.5	>64
**14c**	1	2	>64	>64	>64	>64	0.125	>64
**14d**	4	4	>64	>64	>64	>64	0.5	>64

**Table 3:** Minimum inhibitory concentrations (MIC) of 12 conjugate molecules against clinically important anaerobic and microaerophilic bacterial strains.

Compounds	Anaerobic and Microaerophilic Bacterial Panel, MIC (ug/mL)													
	Gram-Positive							Gram-Negative						Micro- Aerophilic
	Cd ATCC 700057	La ATCC 4356	El ATCC 43055	Gv ATCC 14018	Pm ATCC 23195	Pas ATCC 3553	Pac ATCC 11829	Bf ATCC 25285	Bl ATCC 15707	Fn ATCC 10953	Mm ATCC 35243	Pv ATCC 29303	Vp ATCC 17745	Hp ATCC 43504
**Metronidazole**	≤0.25	>256	0.5	2	>256	0.5	>256	≤0.25	4	≤0.25	8	2	>256	64
**Pretomanid (P)**	2	>32	8	>32	>32	2	>32	16	>32	4	>32	32	>32	16
**Linezolid (L)**	0.5	2	1	0.25	1	0.5	0.06	2	0.5	0.25	0.12	2	0.5	8
**L + P (1:1)**	1	4	2	0.5	2	1	0.25	4	2	1	0.25	4	1	16
**5**	0.06	NA	0.06	0.12	0.25	0.06	1	0.25	0.5	0.06	0.25	0.5	1	-
**8a**	≤0.03	0.5	≤0.03	0.06	0.12	0.06	≤0.03	1	0.25	≤0.03	≤0.03	4	0.25	0.5
**8b**	≤0.03	0.5	≤0.03	0.06	0.25	≤0.03	0.06	4	0.12	≤0.03	0.06	8	0.25	1
**11a**	0.12	0.5	0.06	0.06	0.5	0.06	0.06	2	0.25	0.06	0.06	4	0.25	2
**11b**	0.06	2	0.06	0.12	1	≤0.03	0.12	4	0.25	≤0.03	0.25	8	1	2
**14a**	0.25	1	0.12	0.12	0.5	0.12	0.12	1	0.25	0.12	0.25	16	0.5	2
**14b**	0.5	2	0.12	0.25	1	0.25	0.25	4	0.5	1	0.5	>32	1	8
**14c**	0.06	0.5	≤0.03	0.12	0.5	≤0.03	0.06	0.5	0.25	≤0.03	0.12	8	0.25	1
**14d**	0.25	2	0.12	0.12	1	0.12	0.25	8	0.5	0.12	0.25	16	0.5	1
**16**	4	>32	16	>32	>32	8	>32	>32	>32	>32	>32	>32	>32	16
**19a**	≤0.03	0.25	≤0.06	0.06	0.12	≤0.03	≤0.03	0.12	0.06	≤0.03	0.06	2	0.25	0.25
**19b**	≤0.03	0.5	0.06	0.06	0.12	≤0.03	0.06	1	0.06	≤0.03	0.06	4	0.25	0.25
**19c**	≤0.03	0.25	≤0.03	≤0.03	0.06	≤0.03	≤0.03	0.5	0.12	≤0.03	0.06	2	0.25	0.5

**Table 4:** List of bacterial strains used in the current study.

Panel	Organism	ATCC No.	Testing Facility
Spectrum Panel	*Enterococcus faecalis* (Ef)	700221	HD Biosciences
	*Staphylococcus aureus* (Sa)	292213	
	*Klebciella pneumonia* (Kp)	43816	
	*Acinetobacter baumannii* (Ab)	19606	
	*Pseudomonas aeruginosa* (Pa)	27853	
	*Escherichia coli* (Ec)	25922	
	*Clostrium difficile* (Cd)	43255	
*M. tuberculosis* Isogenic Resistant Mutant Panel	*M. tuberculosis* H37Rv (Wild-Type)	27294	BTTTRI
	*M. tuberculosis* P1 (Pretomanid-R)	Isogenic	
	*M. tuberculosis* P3 (Pretomanis-R)	Isogenic	
	*M. tuberculosis* L1 (Linezolid-R)	Isogenic	
	*M. tuberculosis* L3 (Linezolid-R)	Isogenic	
Anaerobic Bacterial Panel	*Clostridium difficile* (Cd)	700057	Micromyx
	*Lactobacillus acidophilus* (Lc)	4356	
	*Eggerthella lenta* (El)	43055	
	*Gardnerella vaginalis* (Gv)	14018	
	*Peptostreptococcus micros* (Pm)	23195	
	*Porphyromonas asaccharolytica* (Pas)	M3553	
	*Propionibacterium acnes* (Pac)	11892	
	*Bacteroides fragilis* (Bf)	25285	
	*Bifidobacterium longum* (Bl)	15707	
	*Fusobacterium nucleatum* (Fn)	10953	
	*Mobiluncus mulieris* (Mm)	35243	
	*Prevotella bivia* (Pb)	29303	
	*Veillonella parvula* (Vp)	17745	
	*Helicobacter pylori* (Hp)	43504	
